# Serum levels of autoantibodies against C-reactive protein correlate with renal disease activity and response to therapy in lupus nephritis

**DOI:** 10.1186/ar2880

**Published:** 2009-12-11

**Authors:** Christopher Sjöwall, Agneta Zickert, Thomas Skogh, Jonas Wetterö, Iva Gunnarsson

**Affiliations:** 1 Rheumatology/AIR, Clinical and Experimental Medicine, Linköping University, SE-581 85 Linköping, Sweden; 2 Department of Medicine, Rheumatology Unit, Karolinska University Hospital, Solna, SE-171 76 Stockholm, Sweden

## Abstract

**Introduction:**

Serum levels of C-reactive protein (CRP) seldom reflect disease activity in systemic lupus erythematosus (SLE). We have previously shown that autoantibodies against neo-epitopes of CRP often occur in SLE, but that this does not explain the modest CRP response seen in flares. However, we have repeatedly found that anti-CRP levels parallel lupus disease activity, with highest levels in patients with renal involvement; thus, we aimed to study anti-CRP in a material of well-characterized lupus nephritis patients.

**Methods:**

Thirty-eight patients with lupus nephritis were included. Treatment with corticosteroids combined with cyclophosphamide, mycophenolate mofetil or rituximab was started after baseline kidney biopsy. A second biopsy was taken after ≥ 6 months. Serum creatinine, cystatin C, complement, anti-dsDNA, anti-CRP and urinalysis were done on both occasions. Biopsies were evaluated regarding World Health Organisation (WHO) class and indices of activity and chronicity. Renal disease activity was estimated using the British Isles Lupus Assessment Group (BILAG) index.

**Results:**

At baseline, 34/38 patients had renal BILAG-A; 4/38 had BILAG-B. Baseline biopsies showed WHO class III (n = 8), IV (n = 19), III to IV/V (n = 3) or V (n = 8) nephritis. Seventeen out of 38 patients were anti-CRP-positive at baseline, and six at follow-up. Overall, anti-CRP levels had dropped at follow-up (*P *< 0.0001) and anti-CRP levels correlated with renal BILAG (*r *= 0.29, *P *= 0.012). A positive anti-CRP test at baseline was superior to anti-dsDNA and C1q in predicting poor response to therapy as judged by renal BILAG. Baseline anti-CRP levels correlated with renal biopsy activity (*r *= 0.33, *P *= 0.045), but not with chronicity index. Anti-CRP levels were positively correlated with anti-dsDNA (fluorescence-enhanced immunoassay: *r *= 0.63, *P *= 0.0003; *Crithidia luciliae *immunofluorescence microscopy test: *r *= 0.44, *P *< 0.0001), and inversely with C3 (*r *= 0.35, *P *= 0.007) and C4 (*r *= 0.29, *P *= 0.02), but not with C1q (*r *= 0.14, *P *= 0.24). No associations with urinary components, creatinine, cystatin C or the glomerular filtration rate were found.

**Conclusions:**

In the present study, we demonstrate a statistically significant correlation between anti-CRP levels and histopathological activity in lupus nephritis, whereas a baseline positive anti-CRP test predicted poor response to therapy. Our data also confirm previous findings of associations between anti-CRP and disease activity. This indicates that anti-CRP could be helpful to assess disease activity and response to therapy in SLE nephritis, and highlights the hypothesis of a pathogenetic role for anti-CRP antibodies in lupus nephritis.

## Introduction

Systemic lupus erythematosus (SLE) is characterized by multiple organ involvement, by production of a wide range of antinuclear antibodies and by the presence of immune complexes in the inflamed organs [1]. Impaired clearance of cellular debris by the reticuloendothelial system is considered a key event in the initiation and maintenance of SLE. Autoantigens escaping physiological clearance may thus become excessively presented to the adaptive immune system, resulting in loss of peripheral tolerance and occurrence of a multitude of autoantibodies - the waste disposal theory [2]. Antibodies against dsDNA are frequently found both in serum and inflammatory lesions in glomerulonephritis [3]. The circulating levels of anti-dsDNA often correlate with disease activity, and these autoantibodies are presumed to be of pathogenetic importance in lupus nephritis [4-6].

The pentraxins constitute an evolutionarily conserved group of proteins, which are expressed during infection, systemic inflammation or tissue damage and participate in the acute phase response in many species [7]. The pentraxin family includes long pentraxins (such as pentraxin 3, produced by mononuclear cells in response to lipopolysaccharides, IL-1β and TNF) and the liver-derived short pentraxins C-reactive protein (CRP) and serum amyloid P component mainly generated by stimulation with IL-6 [7]. Despite raised levels of IL-6 and extensive systemic inflammation, serum CRP concentrations typically remain low in lupus flares [8], although differences between certain disease manifestations [9] and conflicting data have been reported [10]. The novel *in vitro *finding that IFNα mediates suppression of IL-6-induced CRP expression in human hepatocytes, however, could possibly explain the weak CRP response in SLE flares [11].

CRP has several biological functions that are related to affinity for molecules exposed on bacteria and apoptotic cells/cell debris, such as phosphorylcholine, nucleosomes, and ribonucleoproteins (snRNPs), thereby resembling a primitive form of a natural antibody [12]. In addition, like IgG class antibodies, CRP interacts with cellular Fcγ receptors, thereby facilitating the phagocytic clearance of circulating opsonized material. Activation of the classical complement pathway is considered one of the main physiological functions of CRP. In contrast to IgG-mediated classical activation, however, CRP-mediated activation appears to be essentially limited to the initial stages involving C1 to C4, with less formation of the membrane attack complex [13]. Furthermore, at sufficient concentrations, soluble native CRP may prevent activation of the classical complement pathway on biological surfaces due to consumption of soluble C1q without binding C2/C4 [14].

In line with its role as a scavenger of autoantigens from dead or dying cells, single nucleotide polymorphisms of the CRP gene have been found to associate with low baseline levels of CRP, with production of antinuclear antibodies, and with increased susceptibility to SLE [8]. Furthermore, in two murine lupus models, subcutaneous CRP injections delayed the disease onset, reversed nephritis, and prolonged the survival of the animals - indicating a preventive and disease-modifying role for CRP in SLE [8,13]. Very recently, however, this finding was contradicted by others [15].

The presence of autoantibodies against CRP in lupus was originally described by Frank A Robey and coworkers in 1985 [16]. Later, Bell and colleagues reported a high frequency of autoantibodies against a certain dissociated and tissue-bound form of CRP, recognized as monomeric CRP (mCRP) in SLE, and at lower prevalence rates in subacute cutaneous lupus erythematosus and primary biliary cirrhosis [17]. Since then several groups have confirmed the finding of IgG class autoantibodies against monomeric CRP (anti-CRP) in SLE and in some other rheumatic conditions [18-22]. In addition, the presence of autoantibodies against pentraxin 3 was recently shown in SLE patients [23,24].

In a series of papers, we have demonstrated the strong correlations between anti-CRP antibody level and disease activity as reflected by the SLE disease activity index, anti-dsDNA antibody levels, and complement levels [12]. The anti-CRP assay has been shown to be antigen-specific [17], without false positive results due to the presence of immune complexes [19,25] or antibodies to DNA or nucleosomes [26]. We have also consistently found that most patients with raised anti-CRP antibody levels appear to have a disease phenotype with renal involvement. The latter was recently confirmed by Tan and colleagues, who found elevated anti-CRP in SLE patients - where the antibody levels paralleled disease activity, particularly in individuals with lupus nephritis [27]. The authors also studied renal histopathology and found that anti-CRP antibody levels correlated with tubulointerstitial lesions and the chronicity index, but not with the activity index [27]. The aim of the present study was therefore to compare anti-CRP antibody levels in well-characterized lupus nephritis patients and to seek potential associations with histopathology, renal activity and response to therapy.

## Materials and methods

### Patients

Thirty-eight patients meeting the 1982 American College of Rheumatology classification criteria for SLE [28] were included in the study. All patients had biopsy-proven active lupus nephritis (during the period 1995 to 2006) and participated in a prospective control programme at the rheumatology clinic of Karolinska University Hospital (Stockholm, Sweden). Thirty-four of the 38 patients (89%) were women (mean age, 33.0 years; range, 19 to 61 years) and four patients were men (mean age, 34.8 years; range, 18 to 50 years). Thirty-three out of 38 patients (87%) were Caucasian. The mean duration of SLE was 6.9 years (range, 0 to 34 years). At baseline, 27 patients displayed proliferative nephritis (World Health Organisation (WHO) class III or IV), eight patients showed membranous pattern (WHO class V), and biopsies in three patients were classified as both proliferative and membranous.

The patients were treated in accordance with clinical routine for lupus nephritis [29], including corticosteroids combined with cyclophosphamide intravenously (n = 27) or orally (n = 1), rituximab (n = 6), and mycophenolate mofetil (n = 3). One patient was initially treated with mycophenolate mofetil, but switched to intravenous cyclophosphamide after 3 months. At the timepoint for the first renal biopsy, clinical data, blood samples and urinary samples were collected. Serum samples were kept frozen at -70°C for future analyses. After induction therapy (mean time, 8 months; range, 6 to 15 months), the patients underwent a second renal biopsy and further clinical and laboratory data were collected. Additional data are presented in Table 1.

**Table 1:** Clinical characteristics and laboratory data for patients

	Baseline			Follow-up		
	
	Anti-CRP-positive	Anti-CRP-negative	Mann-Whitney	Anti-CRP-positive	Anti-CRP-negative	Mann-Whitney
Age (years)	30.5 (13.1)	35.0 (11.6)	NS	27.0 (8.2)	35.1 (12.7)	NS
Gender						
Female	16	18		5	29	
Male	1	3		1	3	
Ethnicity						
Caucasian	16	17		5	28	
Iranian Caucasian	0	3		0	3	
Iraqi Caucasian	0	1		0	1	
Asian	1	0		1	0	
Creatinine (μmol/l)	95.3 (43.3)	97.0 (51.0)	NS	78.3 (9.6)	83 (46.2)	NS
Albuminuria (g/day)	1.7 (1.4)	2.7 (2.3)	NS	1.6 (1.3)	0.8 (0.9)	NS
C3 (g/l)	0.47 (0.26)	0.57 (0.24)	NS	0.85 (0.28)	0.80 (0.27)	NS
C4 (g/l)	0.09 (0.06)	0.10 (0.05)	NS	0.14 (0.03)	0.14 (0.07)	NS
C1q (% of normal reference)	57.9 (37.3)	79.0 (36.7)	NS	73.0 (32.4)	83.0 (28.1)	NS
Anti-dsDNA-positive	16	18		3	21	
CLIFT titre, median	200	25	*P *= 0.04	25	10	NS
Renal histopathology						
Class I	0	0		0	1	
Class II	0	0		1	13	
Class III	1	7		1	2	
Class IV	12	7		2	4	
Class III to IV/V	1	2		0	2	
Class V	3	5		2	10	
Activity index	7.0 (3.1)	5.5 (3.5)	NS	3.5 (2.0)	2.7 (3.2)	NS
Chronicity index	2.3 (1.9)	1.4 (2.0)	NS	2.7 (1.0)	2.7 (2.4)	NS
BILAG index						
A	14	20		1	5	
B	3	1		4	14	
C	0	0		0	8	
D	0	0		1	5	
Treatment						
Prednisolone, mean daily dose (mg)	10.1 (10.5)	12.9 (17.8)	NS	14.5 (7.2)	10.6 (5.0)	NS
Mycophenolate mofetil	1	2				
Cyclophosphamide	15	13				
Rituximab	0	6				
Mycophenolate mofetil/cyclophosphamide	1	0				

Data presented as mean (standard deviation) or *n*. BILAG, British Isles Lupus Assessment Group; CLIFT, *Crithidia luciliae *immunofluorescence microscopy test; CRP, C-reactive protein; NS, not significant.

### Renal histopathology

Renal biopsies were performed by percutaneous ultrasonography-guided puncture in accordance with a standard protocol. The renal tissue obtained was staged according to the WHO classification for lupus nephritis [30]. All biopsies were evaluated by light microscopy, immunofluorescence and electron microscopy. The biopsies were graded according to a standardized semiquantitative histological scoring protocol for activity and chronicity indices [31].

### Renal disease activity and response to therapy

Renal disease activity was estimated using the classical British Isles Lupus Assessment Group (BILAG) index [32]. An improvement of at least two grades in the renal domain of BILAG (that is, from A to C or from B to D) at follow-up was required for the patient to be regarded as a responder. The BILAG index was translated into numerical data for correlation analyses (A = 9, B = 3, C = 1 and D = 0) as suggested by Dr David A Isenberg, London (personal communication).

### Laboratory and serological measures

Renal function was monitored by urinalysis (dip-slide procedure), urinary sediment assessment, 24-hour urine albumin excretion, serum creatinine, the glomerular filtration rate assessed by urinary clearance of iohexol according to clinical routine, and cystatin C (turbidimetry).

Analyses of complement component C1q were performed by rocket electrophoresis using polyclonal rabbit anti-C1q (DAKO, Glostrup, Denmark). Levels of C1q were expressed as the percentage of the levels of healthy blood donors (normal range, 76 to 136%). C3 (normal range, 0.70 to 1.3 g/l) and C4 (normal range, 0.13 to 0.32 g/l) were determined by nephelometry.

Assessments of serum IgG anti-dsDNA antibodies were made by the ImmunoCAP fluorescence-enhanced immunoassay (Pharmacia, Uppsala, Sweden), normal range <15 IU/ml, and by the *Crithidia luciliae *immunofluorescence microscopy test (CLIFT) with cut-off titre 1:10.

### Anti-C-reactive protein antibody assay

IgG anti-CRP antibodies were measured with an ELISA as described previously [26]. To avoid systematic errors, samples from patients and controls were always randomly mixed on the microtitre plates and analysed on the same occasion. Anti-CRP antibody levels were expressed as the percentage of a positive reference sample from a SLE patient at flare representing 100 arbitrary units. No differences were apparent considering the groups of men and women in the control material. To exclude the possibility of non-specific binding, each serum was also tested in the same way on uncoated plates.

### Statistics

Figures were prepared in GraphPad Prism (version 4.0; GraphPad Software Inc., San Diego, CA, USA). Correlation analyses were performed using Spearman's rank correlation (SPSS for Windows version 15.0.0; SPSS Inc. (IBM), Chicago, IL, USA), and differences between groups were calculated with the Wilcoxon signed rank test or the Mann-Whitney *U *test (GraphPad). Response to therapy was compared by chi-square analysis using StatCalc (Epi Info version 3.5.1; Centers for Disease Control and Prevention, Atlanta, GA, USA). Two-tailed *P *< 0.05 was considered significant.

### Ethics

Informed consent was obtained from all subjects. The research protocol was approved by the regional ethics committee in Stockholm.

## Results

**Figure 1:**
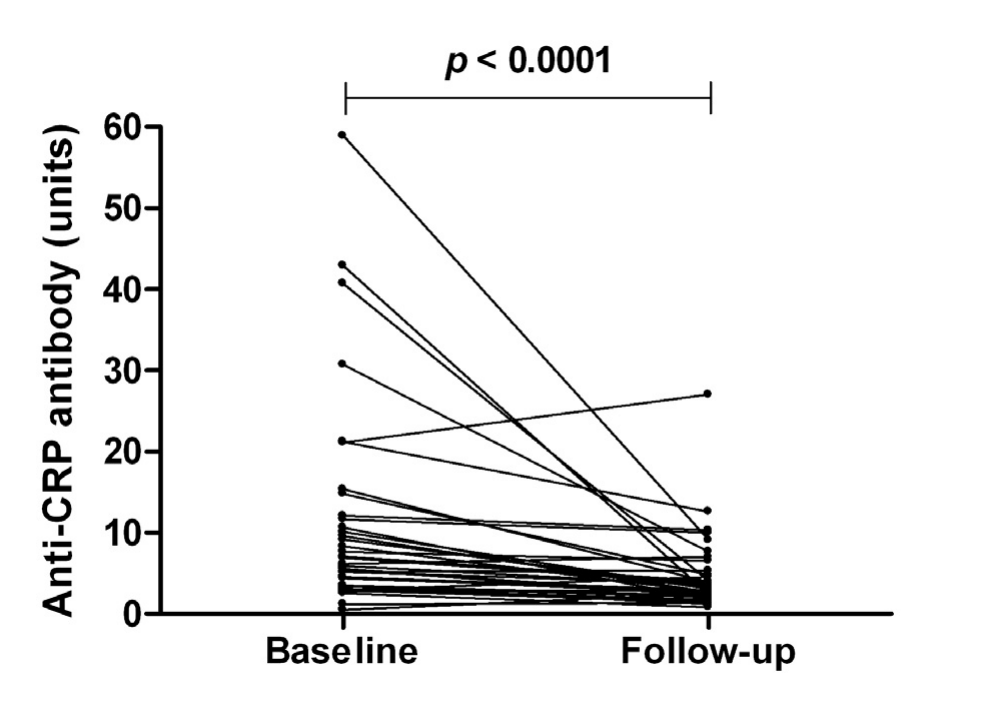
Anti-C-reactive protein antibody levels in systemic lupus erythematosus patients before and after induction therapy. Anti-C-reactive protein (anti-CRP) antibody levels at baseline and follow-up in 37 systemic lupus erythematosus patients (outlier excluded). Paired data were analysed with the Wilcoxon signed rank test.

Anti-CRP antibody levels were determined at baseline and follow-up in each of the 38 patients. The cut-off value for positive reaction was set at 8 units, calculated from the 95th percentile in 100 healthy blood donors (controls). As indicated in Table 1, 17 out of 38 patients (45%) were judged anti-CRP antibody-positive at baseline and six patients (16%) were positive at follow-up. Overall, the anti-CRP antibody levels were significantly reduced at follow-up (*P *< 0.0001) (Figure 1).

**Figure 2:**
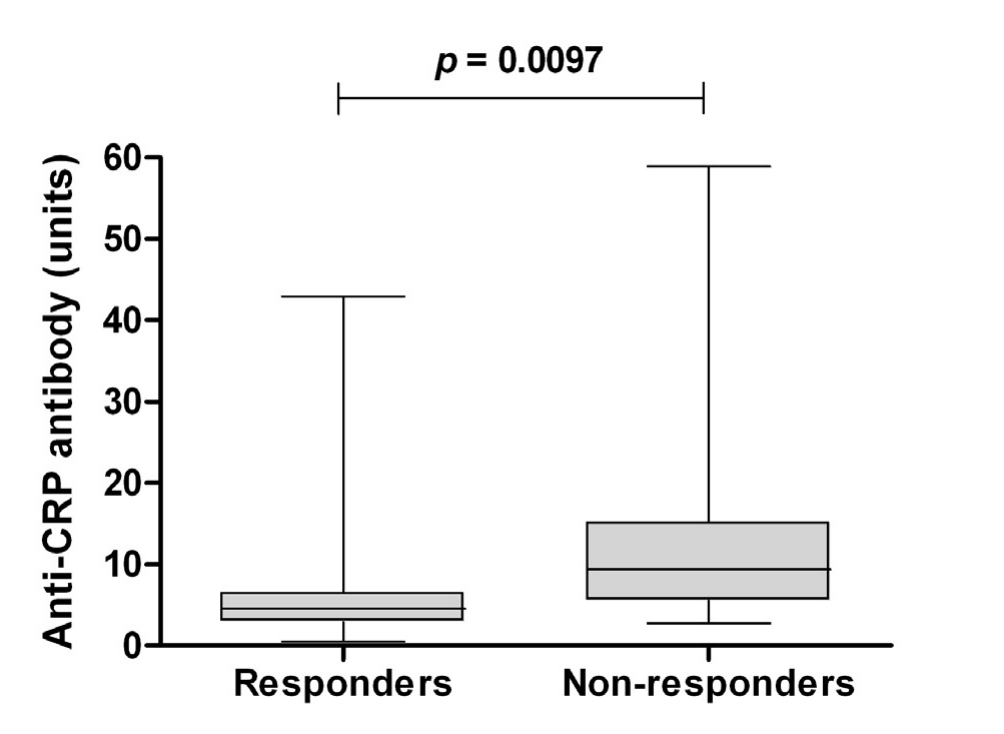
Baseline anti-C-reactive protein antibody levels in responders and non-responders to given therapy. At baseline, there was a highly significant difference in anti-C-reactive protein (anti-CRP) antibody levels between patients that would respond (n = 16) and would not respond (n = 22) to therapy (analysed with Mann-Whitney *U *test). Response to therapy was defined as a renal British Isles Lupus Assessment Group improvement ≥ 2 grades. The limitations extend down from the lowest value and up to the highest. Median value for responders was 4 units, and was 9 units for nonresponders. Boxes show the 25th to 75th percentile, with median values marked inside.

**Figure 3:**
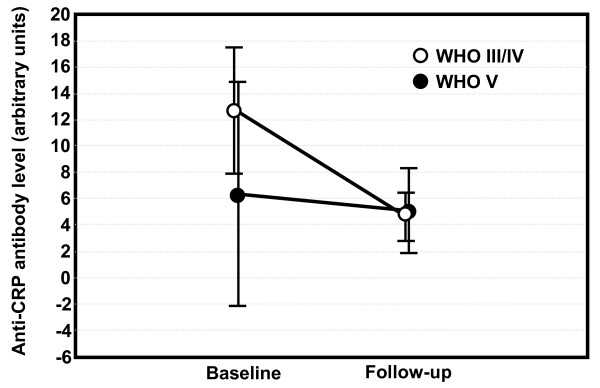
Anti-C-reactive protein antibody levels in patients with proliferative versus membranous lupus nephritis. The levels of anti-C-reactive protein (anti-CRP) antibodies in the 26 patients with proliferative lupus nephritis (World Health Organisation (WHO) class III/IV) were reduced to a greater extent than the eight patients with membranous lupus nephritis (WHO class V), although not statistically significant by the Mann-Whitney *U *test (mean difference -8.3 vs. -1.1 units; *P *= 0.08). The vertical bars denote 95% confidence intervals. The outlier as well as the three patients with class III to IV/V nephritis were not included in this analysis.

Anti-CRP antibody levels were more efficiently reduced in patients who responded to therapy as judged by renal histopathology (data not shown), and baseline anti-CRP levels were significantly higher (*P *= 0.0097) in patients who did not reach a renal BILAG improvement of at least two grades (Figure 2). Neither a baseline lowered C1q (relative risk = 1.58, 95% confidence interval = 0.87 to 2.84) nor a positive anti-dsDNA antibody test as judged by the CLIFT (relative risk = 1.18, 95% confidence interval = 0.42 to 3.26) predicted response to therapy, whereas a positive anti-CRP test did (relative risk = 2.16, 95% confidence interval = 1.20 to 3.89). Response to therapy in relation to anti-CRP, adjusted for C1q and anti-dsDNA antibody status, respectively, is illustrated in Tables 2 and 3. A positive anti-CRP test was thus associated with a poor response to therapy, particularly in patients with normal C1q levels and a positive anti-dsDNA antibody test (CLIFT). Patients with proliferative nephritis (WHO class III or IV) had a greater reduction of anti-CRP antibody levels compared with patients with membranous (WHO class V) nephritis (Figure 3), although this was not statistically significant (*P *= 0.08).

**Table 2:** Renal BILAG response and the C1q/anti-CRP status demonstrated

	BILAG response <2	BILAG response ≥ 2
C1q low		
Anti-CRP-positive	8	2
Anti-CRP-negative	5	5
Relative risk	1.60 (0.80 to 3.20)	
C1q normal		
Anti-CRP-positive	6	1
Anti-CRP-negative	3	8
Relative risk	3.14 (1.14 to 8.64)	

Relative risks presented as chi-square (95% confidence interval). BILAG, British Isles Lupus Assessment Group; CRP, C-reactive protein.

**Table 3:** Renal BILAG response and the anti-dsDNA/anti-CRP status demonstrated

	BILAG response <2	BILAG response ≥ 2
Anti-dsDNA-positive		
Anti-CRP-positive	13	3
Anti-CRP-negative	7	11
Relative risk	2.09 (1.12 to 3.90)	
Anti-dsDNA-negative		
Anti-CRP-positive	1	0
Anti-CRP-negative	1	2
Relative risk	3.00 (0.61 to 14.86)	

Relative risks presented as chi-square (95% confidence interval). BILAG, British Isles Lupus Assessment Group; CRP, C-reactive protein.

**Figure 4:**
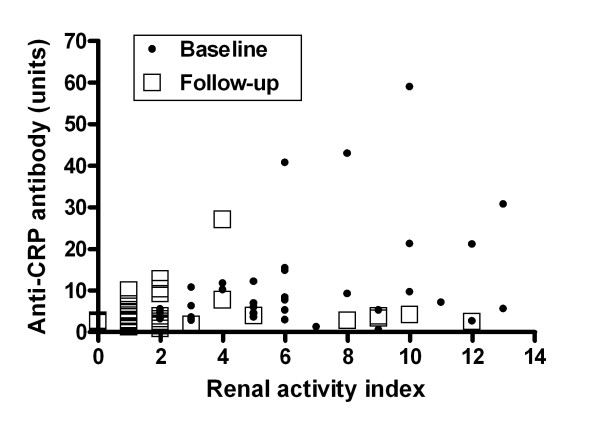
Correlation between anti-C-reactive protein antibody levels and the renal histopathology activity index. Anti-C-reactive protein (anti-CRP) antibody levels at baseline correlated with the renal histopathology activity index according to the description by Austin and colleagues [31] (*r *= 0.33, *P *= 0.045), whereas the association at follow-up did not reach statistical significance (*r *= 0.30, *P *= 0.061). Correlations were analysed with Spearman's rank correlation. Anti-CRP levels in relation to the renal activity index are shown for baseline and follow-up samples, respectively, in 37 patients (outlier excluded).

Of the 17 anti-CRP antibody-positive patients at baseline, 16 patients had received cyclophosphamide and two patients received mycophenolate mofetil (one in combination with cyclophosphamide). One individual increased dramatically in anti-CRP, from 8 units at baseline to 214 units at follow-up. This patient with WHO class IVc nephritis received cyclophosphamide intravenously, but did not respond to therapy and was regarded as an outlier (not included in all analyses). All six anti-CRP antibody-positive patients at follow-up were positive also at baseline, and all had received cyclophosphamide intravenously as induction therapy. Five of these six patients did not respond to therapy as judged by renal histopathology; one patient switched from WHO class IVb to a IIa pattern, but the remaining five patients had a similar or worsened histopathologic picture with increment in the chronicity index. Using the BILAG index, the same five individuals with persistently raised anti-CRP levels did not respond to therapy (renal BILAG improvement ≥ 2 grades). In the whole material, no obvious relation between anti-CRP antibody reduction and the type of induction therapy was found. All six patients receiving rituximab were consistently anti-CRP-negative. As illustrated in Figure 4, anti-CRP antibody levels at baseline showed a moderate but statistically significant positive correlation with the renal biopsy activity index (*r *= 0.33, *P *= 0.045), whereas the association at follow-up did not reach statistical significance (*r *= 0.30, *P *= 0.061). Accumulated anti-CRP data yielded an even stronger correlation with histopathological activity (*r *= 0.37, *P *= 0.0017). No associations between anti-CRP levels and the chronicity index were found (not shown).

**Figure 5:**
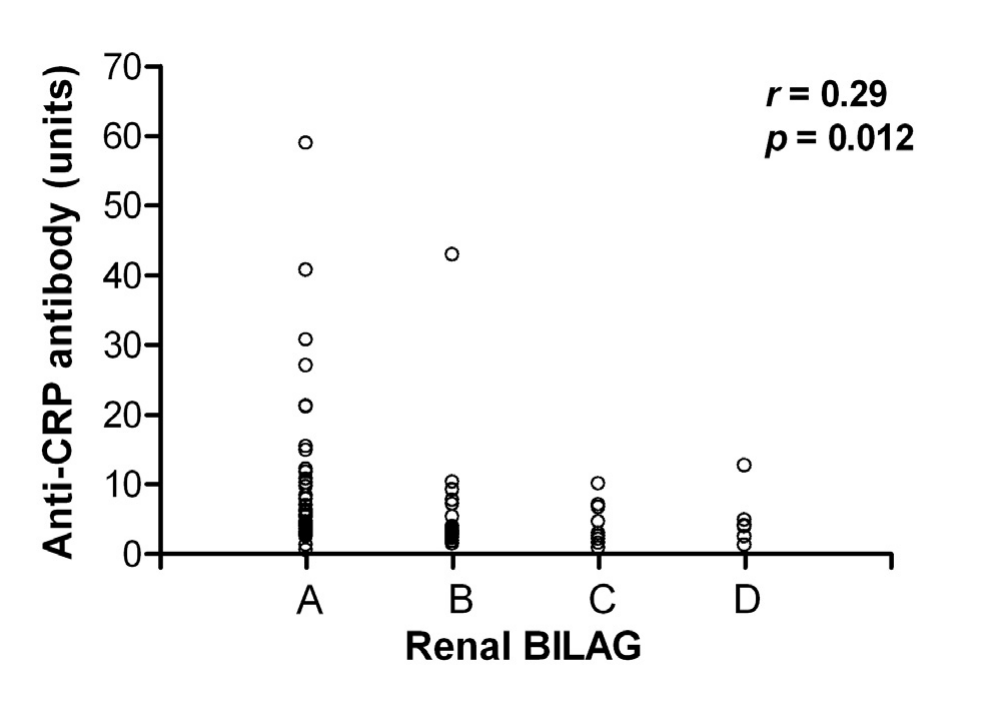
Anti-C-reactive protein antibody level correlation with the renal British Isles Lupus Assessment Group index. Anti-C-reactive protein (anti-CRP) antibody levels correlated significantly with renal disease activity, as assessed by the classical British Isles Lupus Assessment Group (BILAG) index [32] using Spearman's rank correlation. Analysis from baseline and follow-up observations in 37 patients (outlier excluded).

Figure 5 shows the correlation between anti-CRP antibody levels and the BILAG index (*r *= 0.29, *P *= 0.012); when solely anti-CRP-positive samples were included, the coefficient was slightly decreased (*r *= 0.20). Using only anti-CRP-positive/anti-dsDNA-negative samples (as judged by the CLIFT), anti-CRP levels were barely associated with renal BILAG, but the number of observations here were small (*r *= 0.10). The correlation between BILAG and anti-dsDNA antibodies was weaker still, however, measured by the fluorescence-enhanced immunoassay (*r *= 0.04) as well as by the CLIFT (*r *= 0.14); when anti-DNA-negative samples (CLIFT) were excluded, the coefficient was further decreased (*r *= 0.08). Looking at anti-CRP-negative/anti-dsDNA-positive samples (CLIFT), anti-DNA was not significantly associated with renal BILAG. On the other hand, anti-CRP levels correlated with anti-dsDNA antibodies as measured with fluorescence-enhanced immunoassay (*r *= 0.63, *P *= 0.0003) and the CLIFT (*r *= 0.44, *P *< 0.0001). Inverse correlations between anti-CRP and C4 (*r *= 0.29, *P *= 0.02) as well as between anti-CRP and C3 (*r *= 0.35, *P *= 0.007) were observed, while the tendency to an inverse relation between anti-CRP and C1q was not significant (*r *= 0.14, *p *= 0.24). No associations between anti-CRP and urinary components, creatinine, cystatin C or the glomerular filtration rate were found.

## Discussion

In the present study, we demonstrate a moderate but statistically significant correlation between anti-CRP antibody levels and renal biopsy activity index in lupus nephritis. Elevated baseline levels of anti-CRP were also found to be predictive of the therapeutic response as judged by renal BILAG at follow-up. Finally, the present study also confirms previous findings of associations between anti-CRP and lupus disease activity as assessed by common clinical and laboratory disease activity measures, such as complement and anti-dsDNA levels. Although the results of this descriptive study do not allow conclusions regarding nephritogenic properties of anti-CRP antibodies, our data imply that anti-CRP antibody testing is a useful tool to support the clinician's evaluation of disease activity and response to therapy in lupus nephritis.

Distinction of disease activity from organ damage in SLE remains a challenge. Indirect assessment of disease activity such as the BILAG index has proven reliable and sensitive to change [33], but it is time consuming and requires a deft certified clinician. Microscopic examination of a kidney biopsy, with classification and estimation of indices of renal disease activity and chronicity, today offers the best possibility to estimate renal disease and its response to therapy. Biopsy is costly and is associated with considerable risks for the patient, however, and can therefore not be done *ad infinitum*. Laboratory variables such as circulating complement components and anti-dsDNA antibodies can be helpful, but there is an urgent need for additional reliable biomarkers of disease activity in SLE.

We have previously demonstrated that the occurrence of autoantibodies against the tissue-based/monomeric CRP is a common finding in SLE, particularly in patients with nephritis [12], and that anti-CRP antibody levels correlate with SLE disease activity index, anti-dsDNA and complement components [26]. These findings have been confirmed by several groups [18,20-22], although not in a recent study by Kessel and colleagues [34]. According to our experience, the prevalence of positive anti-CRP tests in SLE is 30 to 50% depending on disease activity and disease phenotype [19,25,26]. Since the present study was limited to patients with lupus nephritis, the prevalence of 45% positive anti-CRP antibody tests was slightly below expectation. A higher prevalence rate was reported by Bell and colleagues [17], whereas an appreciably lower frequency was found by Shoenfeld and colleagues [22]. Apart from differences in patient selection and disease phenotype (that is, renal involvement), the diverging results in these studies [18,20-22,27] may be due to differences in methodological details regarding anti-CRP antibody analysis. Contrasting to most other studies, we refrain from the use of BSA to block nonspecific IgG-binding in our anti-CRP antibody assay, since serum antibodies to BSA (and other dietary proteins) are common and may thus affect the results, regardless of whether or not BSA is also included in the dilution buffer [35].

In a recent study by Tan and colleagues, positive correlations were reported regarding anti-CRP antibody levels and chronic renal histology features such as tubular atrophy, interstitial fibrosis and the chronicity index score, as well as regarding disease activity assessments (that is, interstitial inflammation and the SLE disease activity index) [27]. In contrast to the current study, however, anti-CRP levels were not associated with the renal activity index [27]. Our previous findings [25,26], as well as the results from the present study, support the notion that anti-CRP primarily reflects disease activity rather than chronicity, severity or organ damage. The connection with renal involvement is clear cut, but seems to be stronger in WHO class III or IV than in membranous nephritis (Figure 3). In this context, very interestingly, the presence of surface-bound CRP has been demonstrated in the renal mesangium and in glomerular capillary walls in specimens from patients with lupus nephritis [36]. Since CRP was found to co-localize with IgG, it is probable that this actually represent immune complexes consisting of mCRP-anti-CRP. Further studies on this interesting matter are underway.

During induction therapy, anti-CRP appears to behave similarly to anti-dsDNA antibodies [26,37], but differently from autoantibodies to SS-A/Ro and SS-B/La [25] and cardiolipin (unpublished data). It has been hypothesized that anti-CRP antibodies could play a role in lupus-related atherosclerosis [38]. Although Figueredo and colleagues reported a weak association between anti-CRP and anti-phospholipid antibodies in SLE and non-SLE patients, however, they found no association with vascular events or foetal loss [21]. Neither did we find, in our study of non-lupus patients with acute coronary syndrome, any raised anti-CRP levels compared with the age-matched controls that were anamnestically healthy and without medication [39].

The growing interest for mCRP has highlighted its importance in several disease states [40-42] and revealed bioactivities *in vitro *and *in vivo *regarding elimination of immune complexes [43], interaction with the complement system [14,44,45], affinity for different Fcγ receptors [46], and proinflammatory effects on platelets [47] and blood lipids [48]. Given all of these mCRP-mediated biological effects, anti-CRP antibodies may participate in the pathogenesis of lupus nephritis and several mechanisms could be hypothesized. One possibility is that native CRP dissociates into mCRP as it binds to nuclear structures planted on the renal glomerular basement membrane [6,49] due to impaired waste disposal [2]. Similar to anti-dsDNA antibodies [50], anti-CRP antibodies may possibly form *in situ *renal immune complexes, which initiate or amplify the tissue inflammation [36]. Further, if the tissue microenvironment becomes acidic due to inflammation, CRP dissociates to mCRP, which may enhance binding of circulating soluble immune complexes to phagocytic Fcγ receptors [12,43] and constitute a vicious circle.

## Conclusions

We have demonstrated a statistically significant correlation between anti-CRP antibody levels and renal biopsy activity index in patients with lupus nephritis. Anti-CRP antibody levels have previously been found to correlate with disease activity, but the present study is the first to show an association with renal disease activity assessed with the BILAG index. In addition, the study suggests that a positive anti-CRP antibody test is superior to anti-dsDNA antibodies and C1q in predicting poor response to therapy in lupus nephritis as judged by renal BILAG.

## Abbreviations

BILAG: British Isles Lupus Assessment Group; BSA: bovine serum albumin; CLIFT: *Crithidia luciliae *immunofluorescence microscopy test; CRP: C-reactive protein; dsDNA: double-stranded DNA; ELISA: enzyme-linked immunosorbent assay; IFN: interferon; IL: interleukin; mCRP: monomeric C-reactive protein; SLE: systemic lupus erythematosus; TNF: tumour necrosis factor; WHO: World Health Organisation.

## Competing interests

The authors declare that they have no competing interests.

## Authors' contributions

CS contributed to the original idea, laboratory work, interpretation of data and manuscript writing. AZ contributed to patient characterization, acquisition of data and statistics. TS contributed to the original idea, interpretation of data and manuscript writing. JW contributed to interpretation of data, statistics and manuscript writing. IG contributed to patient characterization, acquisition of data and manuscript writing.
